# Oxygen environment and islet size are the primary limiting factors of isolated pancreatic islet survival

**DOI:** 10.1371/journal.pone.0183780

**Published:** 2017-08-23

**Authors:** Hirotake Komatsu, Colin Cook, Chia-Hao Wang, Leonard Medrano, Henry Lin, Fouad Kandeel, Yu-Chong Tai, Yoko Mullen

**Affiliations:** 1 Division of Developmental and Translational Diabetes and Endocrinology Research, Department of Diabetes and Metabolic Researches, Beckman Research Institute of City of Hope, Duarte, California, United States of America; 2 Department of Electrical Engineering, California Institute of Technology, Pasadena, California, United States of America; Virginia Commonwealth University, UNITED STATES

## Abstract

**Background:**

Type 1 diabetes is an autoimmune disease that destroys insulin-producing beta cells in the pancreas. Pancreatic islet transplantation could be an effective treatment option for type 1 diabetes once several issues are resolved, including donor shortage, prevention of islet necrosis and loss in pre- and post-transplantation, and optimization of immunosuppression. This study seeks to determine the cause of necrotic loss of isolated islets to improve transplant efficiency.

**Methodology:**

The oxygen tension inside isolated human islets of different sizes was simulated under varying oxygen environments using a computational *in silico* model. *In vitro* human islet viability was also assessed after culturing in different oxygen conditions. Correlation between simulation data and experimentally measured islet viability was examined. Using these *in vitro* viability data of human islets, the effect of islet diameter and oxygen tension of the culture environment on islet viability was also analyzed using a logistic regression model.

**Principal findings:**

Computational simulation clearly revealed the oxygen gradient inside the islet structure. We found that oxygen tension in the islet core was greatly lower (hypoxic) than that on the islet surface due to the oxygen consumption by the cells. The hypoxic core was expanded in the larger islets or in lower oxygen cultures. These findings were consistent with results from *in vitro* islet viability assays that measured central necrosis in the islet core, indicating that hypoxia is one of the major causes of central necrosis. The logistic regression analysis revealed a negative effect of large islet and low oxygen culture on islet survival.

**Conclusions/Significance:**

Hypoxic core conditions, induced by the oxygen gradient inside islets, contribute to the development of central necrosis of human isolated islets. Supplying sufficient oxygen during culture could be an effective and reasonable method to maintain isolated islets viable.

## Introduction

Pancreatic islet transplant is an effective treatment for type 1 diabetes (T1D), a disease in which the immune system induces insulin depletion by specifically attacking insulin producing beta cells within pancreatic islets [1, 2]. Various forms of insulin injection have been developed as treatments for diabetes; however, they do not solve the underlying destruction of isået cells. Additionally, insulin treatments are associated with inadequate blood glucose control and lethal hypoglycemic incidence [3, 4]. Transplantation of the whole pancreas is a radical approach to free the patient from insulin injections; however, there is a shortage of suitable donor pancreata. Another treatment is islet transplantation, in which islets containing insulin producing beta cells are isolated from the pancreas and transplanted into the liver via the portal vein [5, 6]. Unlike whole pancreas transplantation, this procedure uses a minimally invasive surgery that requires a shorter hospital stay and is associated with fewer complications. With the advancement of regenerative medicine, the generation of islet-like cell clusters may be exploited as a source of donor islets for transplantation [7–9], which can be achieved easier than the generation of whole pancreas.

Current islet transplantation requires a large number of islets to achieve insulin-independence, and 2–3 donor pancreata for each recipient are often required to succeed. Isolated islets start to die during culture prior to transplantation, and it is believed that more than a half of transplanted islets do not engraft after transplantation. Attributable reasons for islet cell death include inflammation, toxic immune products, and hypoxia [10–14]. Because the islet is a cluster consisting of an average of 2000 cells [15], the oxygen and nutrient demands of these cells in culture or transplant site would be different than single cells. Necrosis of cells rarely happens in single cell culture; however, central necrosis of the islet is commonly detected during culture. We previously showed that hyperoxic culture maintains viable islet mass and better beta cell function [16]. Because oxygen depletion is considered the primary cause of both central necrosis and the malfunction of isolated/transplanted islets, several oxygenation methods have been introduced for *in vitro* and *in vivo* use [17–22]. However, the relation between lack of oxygen and cell viability has not been fully clarified, which represents a barrier to successfully using transplanted islets as a therapy for T1D. In this study we hypothesize that hypoxia plays a pivotal role in islet cell death. To test this hypothesis, we performed *in silico* simulations and obtained *in vitro* islet viability data to clarify the relationship between oxygen and islet death.

## Materials and methods

### Human islets

Human islets were isolated from organ donors allocated by United Network for Organ Sharing (UNOS). Isolated islets were provided by Southern California Islet Cell Resources (SC-ICR) Center of City of Hope following the Standard Operation Procedures approved by Institutional Review Board and FDA [23]. None of the donors were from a vulnerable population, and all donors or next of kin provided written informed consent that was freely given.

### Human islet culture under different oxygen concentrations

Islets were cultured using a 24-well plate at a concentration of 250 IEQ/well in a cell culture insert (PICM01250, EMD Millipore, Billerica, MA, USA). IEQ is the standard unit used to express the estimate of islet number [24]. Cells were maintained in 800 μL of RPMI1640 medium (Life Technologies, Carlsbad, CA, USA) containing 5 mmol/L glucose and 10% heat inactivated fetal bovine serum (FBS, Atlanta Biologicals, Lawrenceville, GA, USA) at 37°C under seven different oxygen concentrations (1, 10, 21, 35, 50, 75, and 95% oxygen plus 5% CO_2_ and N_2_) in Modular Incubator Chambers (Billups-Rothenberg, San Diego, CA, USA). Oxygen toxicity experiments were performed using three day cultures; islet survival assessment was performed using seven day cultures.

### Single cell volume calculation of human islets

**Fig 1:**
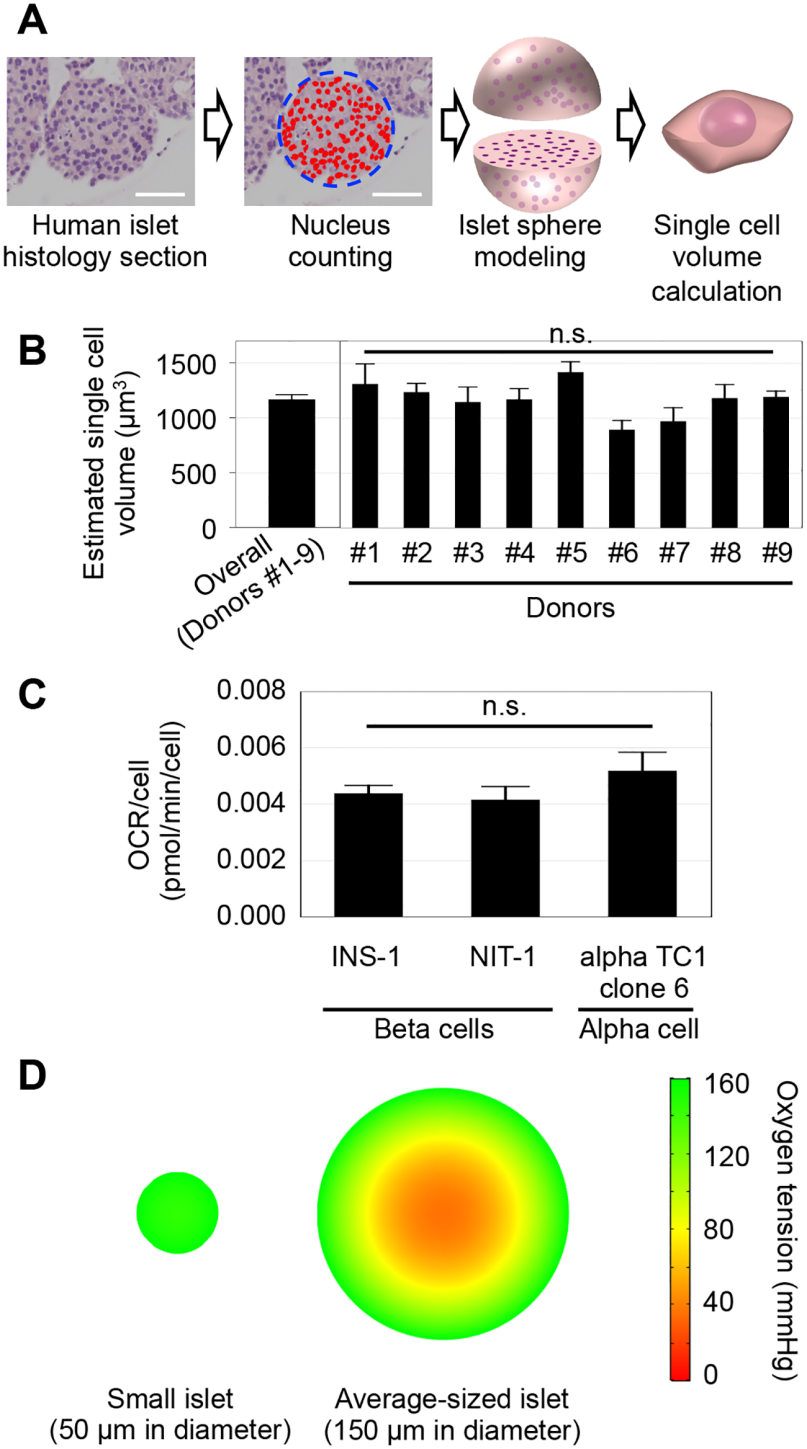
Oxygen simulation reveals the hypoxic core of isolated islets in culture. (A) Method of the single cell volume calculation of a human islet. Automated counting of nuclei in an islet cross section was performed using human islet histology sections stained by hematoxylin and eosin. Islet volume was estimated by islet sphere modeling, and the number of nuclei in the islet was estimated. The islet volume divided by the number of nuclei gives the volume of a single cell volume in the islet. Scale bar: 50 μm. (B) Three islets from each islet donor (#1–9) were examined, and no significant difference in single cell volume was seen (p = 0.08091~1.0, by multiple comparison using Kruskal-Wallis test). The average of the estimated single cell volume obtained from nine donors was 1166.5 μm^3^. (C) OCRs of beta and alpha cells, the dominant population in islets, were measured and used as parameters in simulations. (D) Computational simulation in a 21% oxygen culture environment was performed. Simulation of an islet 150 μm in diameter shows an internal oxygen gradient, whereas simulation of a small islet 50 μm in diameter shows relatively homogeneous oxygen distribution.

Isolated human islets were fixed in 10% formaldehyde (Sigma-Aldrich, St. Louis, MO, USA) for 10 minutes followed by embedding in 3% agarose (Sigma-Aldrich). Embedded islets were incubated again in 10% formaldehyde for 48 hours to prepare paraffin blocks for histology sectioning. Histology sections were stained with hematoxylin-eosin for assessment. For the islet sphere modeling, three circle-shaped islets were randomly selected from each donor islet, and islets from 9 donors were analyzed. The average diameter of these islets was 67 μm (ranging from 40–107 μm), smaller than the general islet size. Because the islet size does not affect the single cell volume, we used smaller islets that were completely circle-shaped for the sphere modeling. Islet sphere modeling was performed to calculate the single cell volume (Fig 1A). Briefly, to calculate the single cell volume, the estimated islet volume was divided by the number of cells in the islet sphere. To estimate number of cells in an islet sphere, the number of nuclei in the islet cross section was obtained using the cellSens 1.12 platform (OLYMPUS, Tokyo Japan).

### Cell culture for measurement of oxygen consumption rate (OCR) in a single cell

A mouse beta cell line (NIT-1), rat beta cell line (INS-1), and mouse alpha cell line (alpha TC1 clone 6) (American Type Culture Collection, Manassas, VA, USA) were cultured in Dulbecco’s modified eagle medium (DMEM, Life Technologies) containing 10% FBS at 37°C in a tissue culture incubator under air plus 5% CO_2_. Cells (2 × 10^4^ cells per well) were seeded in Seahorse XF24 Cell Culture Microplates (Agilent Technologies, Santa Clara, CA, USA) and maintained in culture until cells grew to 70% confluency. Basal OCR was measured six times each for 30 minutes consecutively, and the average was calculated. After OCR measurements, cells were trypsinized, and the cell number was determined for each well. The basal OCR was normalized to the cell number in the well. OCR measurements were performed in octuplicate for each test sample.

### Computational simulation of oxygen tension inside of isolated islets

We simulated the oxygen tension inside islets placed under normal cell culture condition (air containing 21% O_2_, 5% CO_2_, and 74% N_2_), as well as an oxygen environment ranging from 1% to 95% oxygen at 1 atm. Cell volume and OCR of a single islet cell (calculated as above) were used for the simulation. Other parameters used for simulation are shown in Table 1.

**Table 1:** Parameters for islet oxygen simulation.

Description	Abbreviation	Values	References
Diffusivity O_2_ in Islet	*D*	$2.1\times 10^{-9}\;\left[\frac{m^{2}}{s}\right]$	[25]
Solubility O_2_ in Islet	*H*	$1.7\times 10^{-1}\;\left[\frac{mol\;O_{2}}{atm\cdot m^{3}}\right]$	[25]
Cell density	*ρ*	$8.6\times 10^{14}\;\left[\frac{cells}{m^{3}}\right]$	-
O_2_ consumption rate (OCR)	*k*	$7.6\times 10^{-17}\;\left[\frac{mol\;O_{2}}{cell\cdot s}\right]$	-
Michaelis O_2_ constant	*K*_*M*_	$1\times 10^{-3}\;\left[\frac{mol\;O_{2}}{m^{3}}\right]$	[26, 27]
Islet diameter	*d*	(25, 300) × 10^−6^ [*m*]	-
Ambient oxygen tension	*P*_0_	(0.01, 0.95) [*atm*]	-
Oxygen tension within islet	*P*	(calculated) [atm]	-

The finite-element simulation used COMSOL 5.0 (COMSOL, Los Angeles, CA, USA) based on the steady-state reaction-diffusion equation:

$$H\cdot D\cdot \nabla^{2}\left[P\right]=Q$$ (1)

where Q represents the oxygen consumption governed by Michaelis-Menten type kinetics:

$$Q=\rho \cdot k\cdot \frac{P}{P+\frac{K_{M}}{H}}\cdot \delta (\text{P}>0)$$ (2)

Note: ∇^2^ denotes the Laplace operator. (P > 0) is a conditional statement, returning 1 if true and 0 if false. The conditional statement was necessary for convergence of the numerical solution because it prevents oxygen partial pressure from becoming negative, which could otherwise cause instability due to the Michaelis-Menten denominator, $P+\frac{K_{M}}{H}$, approaching 0. Simulations were carried out over a spherical domain defined by the islet diameter with the surface held at the prescribed ambient oxygen tension.

Michaelis-Menten kinetics were used to model the oxygen uptake rate, which approximates cellular metabolism under different oxygen tensions. The form of the equation captures the asymptotic behavior of oxygen uptake rate to a maximum rate *k* when oxygen partial pressure is above *K*_*M*_; when the oxygen partial pressure is below *K*_*M*_ the oxygen uptake rate is proportional to the available oxygen. Because the *K*_*M*_ for beta cells has not been experimentally determined, we used a value similar to that of the mitochondria, consistent with other work modeling the islet [25]. Cells were considered to be uniformly distributed over the spherical islet. Therefore, oxygen uptake rate was treated on a per-volume basis. Finally, the environmental oxygen tension experienced by cells was fixed at the islet periphery.

### Islet viability assay

Islets were stained with fluorescein diacetate (FDA) and propidium iodide (PI) in a 96-well plate to stain viable cells (green) and dead cells (red), respectively [28]. Multiple images were assembled into a single image covering the entire well area. The positive area stained by each dye was measured separately using the cellSens software platform (OLYMPUS). The following formula was used to calculate the viability of each islet:

Viability(%)=100−[(Area_PI/(Area_FDA+Area_PI)*100)] (3)

A total of 1278 human islets from four different donors were used for the analytical plots showing the relationships among islet viability, oxygen tension in culture media, and islet diameter.

### Oxygen toxicity measured by viability assessment

Isolated human islets were filtered using 70 μm cell strainers (Thermo Fisher Scientific, Hampton, NH, USA). Filtered islets were cultured for three days in a 1, 10, 21, 50, 75, and 95% oxygen environment, respectively. Viability was assessed using FDA/PI staining. Islets 40–60 μm in diameter were analyzed in the assembled single image covering the entire well, and an average of 253 islets (198–373 islets) from three different islet batches was cultured in each oxygen setting.

### Absolute oxygen tension in media under various oxygen settings

The absolute oxygen tension in media after culturing islets for 24 hours under 10, 21, 35, and 50% oxygen at 1 atm was measured at the bottom of each well using an oxygen microsensor (Unisense, Aarhus N, Denmark) [16].

### Data analysis

Data for the single cell volume analysis and OCR calculation was analyzed using JMP 9.0.0 (SAS Institute, Cary, NC) and reported as mean ± standard error (SEM). For statistical comparison, nonparametric Wilcoxon tests were performed for single cell volume analysis, and Student’s t-tests were performed for OCR analysis. p < 0.05 denotes statistical significance.

Islet viability data for a three-dimensional (3D) scatterplot and the second order curve of best fit was generated using the MATLAB software platform (Natick, MA, USA). To investigate the effects of islet diameter and oxygen tension on islet viability, logistic regression analyses were performed modeling islet survival as the dependent variable. The islet was considered alive if only more than 95% of the islet volume is viable. The regression estimate of islet diameter and oxygen tension was then used to calculate the predicted islet survival probabilities in different levels of oxygen tension (50, 150, 250, and 350 mmHg) and islet diameter (20–650 μm). Donor-specific predicted survival probabilities were also obtained by including donor IDs in the model. R Statistical Software (version 3.3.2, R Foundation for Statistical Computing, Vienna, Austria) was used to perform these analyses.

## Results

### Oxygen simulation in isolated cultured islets

The volumes of single cells in human islets were calculated using area analysis of islet histology sections. Because the islet shape is close to a sphere, the calculation of single cell volume in an islet was performed using a model of a perfect sphere (Fig 1A). The average estimated volume for a single cell was 1166.5 μm^3^, as calculated using islets obtained from nine donors (Fig 1B). This value was used to calculate the cell density in an islet (Table 1). Because beta and alpha cells are the dominant cells in human islets [29, 30], the OCR obtained from beta and alpha cell lines were used for the simulation. The measured OCR was 4.38 × 10^−3^ pmol/min/cell in a single INS-1 cell (a beta cell line), similar to 4.12 × 10^−3^ pmol/min/cell measured in a single NIT-1 cell (another beta cell-line). The measured OCR was 5.18 × 10^−3^ pmol/min/cell in an alpha TC1 clone 6 cell (an alpha cell-line) (Fig 1B). Because there were no significant differences in OCRs among three cell lines, an average of these OCRs was used for the simulation (Table 1). Using these parameters, computational simulation was carried out in culture under 21% oxygen, the condition most commonly used for islet and cell culture (Fig 1D). An average sized human islet measuring 150μm in diameter exhibited an oxygen gradient with oxygen tension decreasing inside. On the other hand, smaller islets measuring 50μm in diameter exhibited relatively homogeneous oxygen distribution within the islet.

### High oxygen damages islet cells

**Fig 2:**
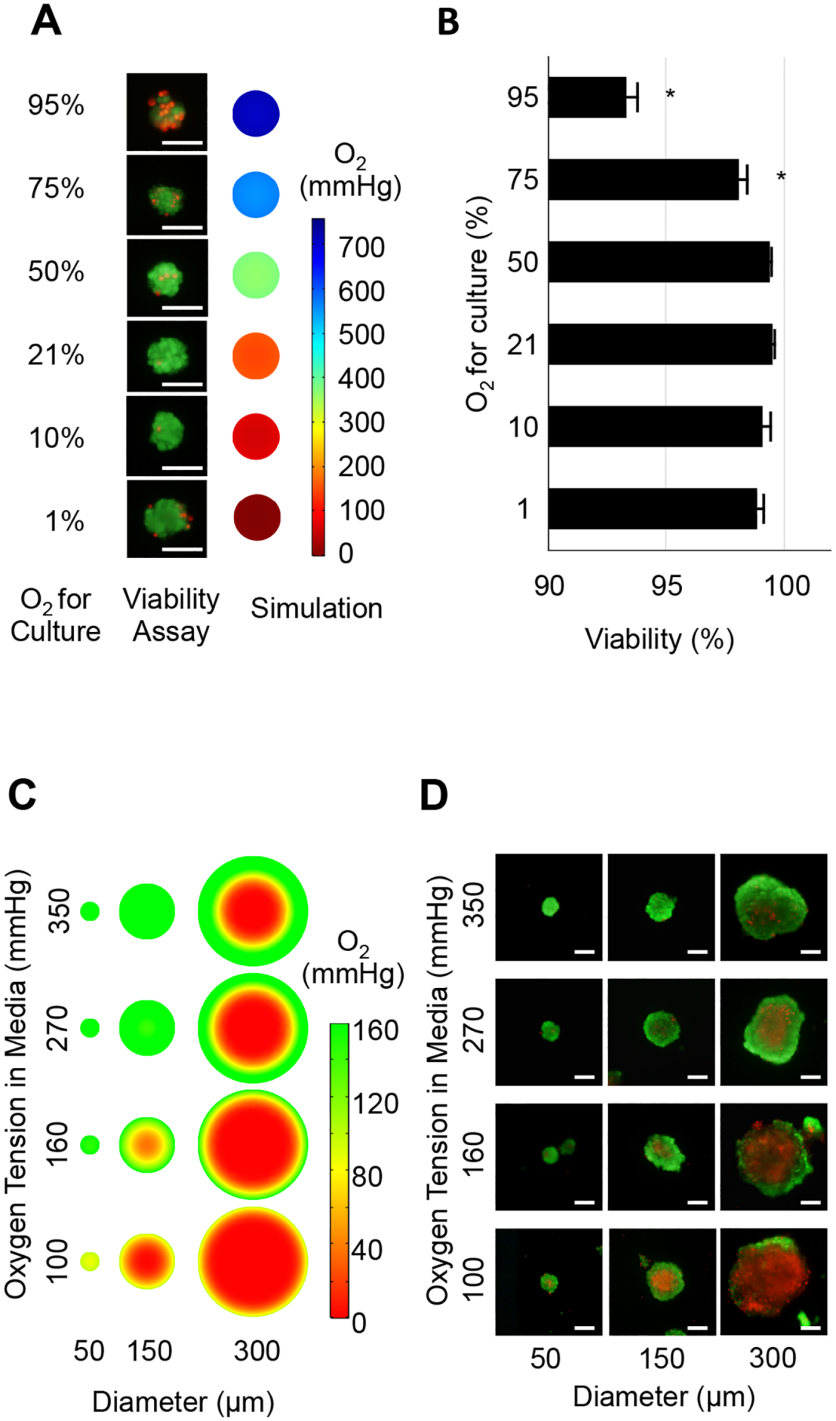
Simulated oxygen in islet and actual viability data in human islets. (A) Representative *in vitro* viability assay and *in silico* oxygen simulation in small islets (<50 μm) under different oxygen culture environments. Viability assay showed dead cells (red fluorescence) in islets cultured under 1, 75, and 95% oxygen culture. Simulation demonstrates uniform distribution of oxygen in the small islet structure. Scale bar: 50 μm. (B) Analysis of viability data indicates that 21% oxygen culture showed highest viability. Oxygenated culture under 75 and 95% demonstrated significantly lower viability compared to that under 21% oxygen (p<0.05), indicating that oxygen above 75% is toxic for islet cells. (C) Oxygen simulation in human islets cultured in oxygen environments between 100, 160, 270, and 350 mmHg. These values correspond to the pO_2_ measured in islet culture media equilibrated in 10, 21, 35, and 50% oxygen, respectively. The simulation revealed a hypoxic core in large islets and hypoxic culture; however, hyperoxic culture reduced the hypoxic area. (D) Representative *in vitro* viability in human isolated islets cultured for 7 days. Large islet diameter and low oxygen culture synergistically induced large central necrosis. The *in vitro* data closely parallels the simulation data, suggesting that the oxygen tension decrease is the primary cause of central necrosis during islet culture. Scale bar: 50 μm.

The simulation showed a hypoxic core in average sized islets (Fig 1D). Hypoxia at the islet core may be prevented by hyperoxic culture that maintains higher oxygen tension throughout the islet. However, higher oxygen levels are non-physiological and known to be toxic to cells [31–33]. Therefore, we first examined the effect of oxygen toxicity by testing viability of small islets measuring 40–60μm in diameter. As demonstrated by oxygen simulation, these small islets have a uniform oxygen distribution throughout the islet (Fig 1D). Dead cells were observed in islets cultured under 1, 75, and 95% oxygen (Fig 2A), whereas viability was highest in 21% oxygen culture (Fig 2B). Compared to the 21% oxygen culture, 75 and 95% oxygen culture showed significantly lower viability (p<0.05). These results indicate that the exposure of islet cells to non-physiologically high oxygen, 75% and higher, is toxic. Low islet viability under 1% oxygen likely indicates an insufficient oxygen supply.

### Hypoxia leads to islet necrosis *in silico* and *in vitro*

To test our hypothesis that hypoxia is the primary cause of central necrosis in cultured islets, the correlation between hypoxia and cell necrosis in the islet core was examined using *in silico* simulation and *in vitro* assessment of cell viability (Fig 2C and 2D). Because high oxygen (>75%) was toxic (Fig 2A and 2B), simulation and *in vitro* experiments were performed using islets cultured between 10 and 50% oxygen. Because the oxygen tension in culture media surrounding the islets is affected by the medium depth and oxygen consumption by cultured islet cells, the absolute oxygen tension measured in the culture media was used for this simulation [16]. The oxygen tension measured in culture media was 100, 160, 270, and 350 mmHg when islets were cultured under 10, 21, 35, and 50% oxygen environment, respectively [16]. Simulation revealed the presence of a hypoxic core in the islets; however, the size of hypoxic core was reduced when islets were placed in hyperoxic (270 and 350 mmHg) culture (Fig 2C). Simulation also revealed that smaller islets (50 and 150 μm) had an increased oxygen tension in the core (Fig 2C).

Next, *in vitro* viability was assessed using human islets. Smaller islets showed less dead area compared to the larger islets. Also, islets cultured in higher oxygen demonstrated less dead area than those cultured in the lower oxygen level when compared to islets of the same size. These *in vitro* data closely corresponded with our oxygen simulation data, supporting the hypothesis that the decrease of oxygen tension is the primary cause of central necrosis in cultured islets.

### Islet viability as a function of islet diameter and medium oxygen tension

**Fig 3:**
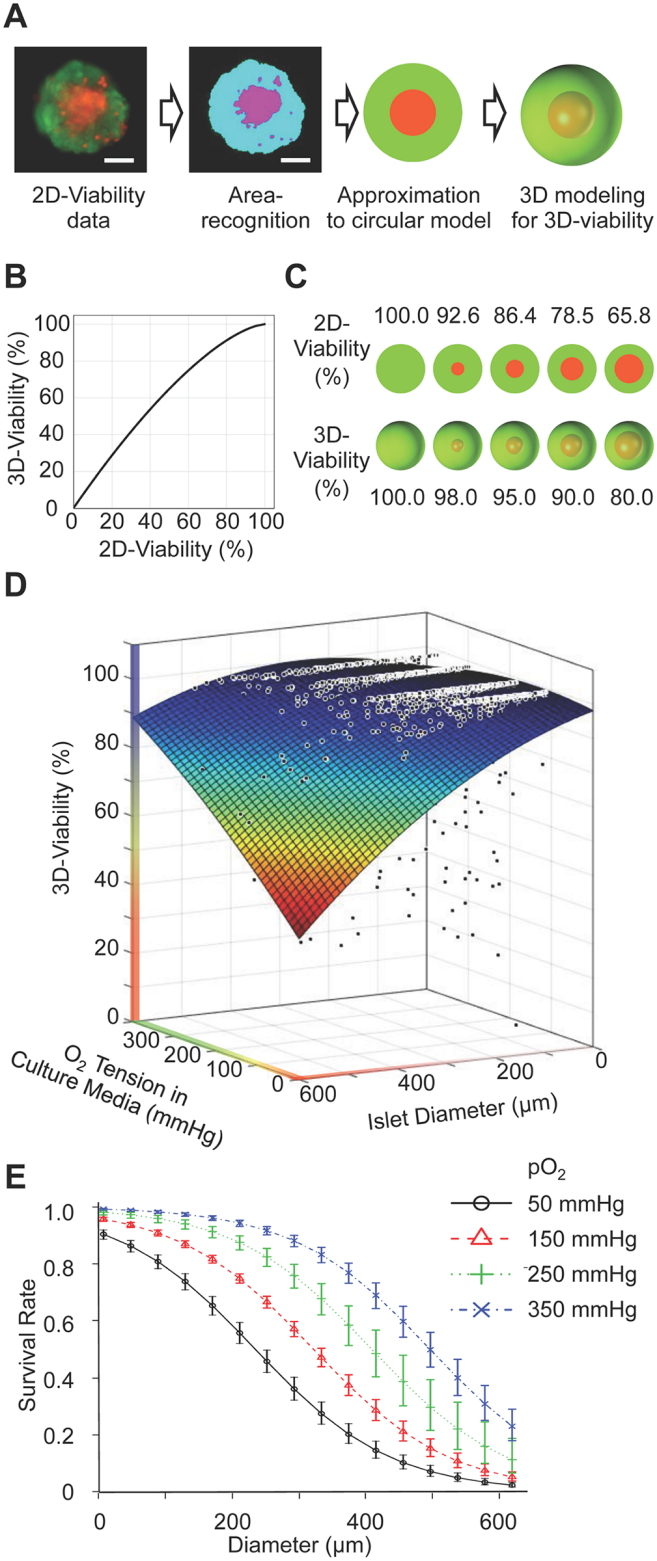
Islet viability is closely related with medium oxygen tension and islet size. (A) 2D viable assay image was converted to 3D to estimate the dead/live volume in the islet. 3D modeling assumed that the islet shape is spherical and that cell death in the islet core occurs concentrically. (B) Relations between 2D and 3D viability data. Scale bar: 50 μm. (C) Representative islet 2D images and corresponding 3D images at specific viabilities. (D) 3D viability plots of human islet of different size cultured in different oxygen environments. Large islet diameter and low oxygen culture media induced low viability. A total 1278 human islets from four different donors were analyzed and plotted. (E) Predicted survival probabilities at different oxygen tension levels were calculated. Error bars indicate the standard error of the prediction estimates. An islet is defined as alive if it has more than 95% 3D-viability. Survival rate declines with increasing islet size, whereas survival rate increases with increasing pO_2_.

Because the computational oxygen tension simulation showed a close relationship between the size of the hypoxic core and the number of dead cells in islets (Fig 2C and 2D), we attempted to clarify the relations among islet viability, medium oxygen tension, and islet diameter. Conventionally, islet viability is assessed using two-dimensional (2D) data obtained using a microscope. However, the oxygen gradient and the patterns of cell death do not occur in a flat plane. Therefore, 2D cell viability was converted to 3D viability to estimate the dead and live cell volume in the islet cluster (Fig 3A). After both alive and dead areas were captured using an automated method [16], the data was converted to a 2D circular model under the assumption that the islet shape is a sphere and the cell death in the islet core occurs concentrically. Finally, the circular model was converted to a 3D spherical model. In this model, the percentage of the dead core area A_%Dead_ to the whole islet area is described as follows:

$${\text{A}}_{\text{\%Dead}}=\frac{r^{2}}{R^{2}}\times 100\text{\%}$$ (4)

where *r* is the radius of the dead core and *R* is that of the islet. Similarly, the percentage of the dead core volume V_%Dead_ to the whole islet volume is calculated as follows:

$${\text{V}}_{\text{\%Dead}}=\frac{r^{3}}{R^{3}}\times 100\text{\%}$$ (5)

Because the viability is defined as the ratio of the viable cells to the whole islet (V_%Viable_), the viability measured by 2D (A_%Viable_ = 100% − A_%Dead_) can be converted to a 3D viability (V_%Viable_ = 100% − V_%Dead_) as follows:

$${\text{V}}_{\text{\%Viable}}=100\text{\%}-\frac{{\left(100\%-{\text{A}}_{\text{\%Viable}}\right)}^{\frac{3}{2}}}{{\left(100\%\right)}^{\frac{1}{2}}}$$ (6)

The relationship between 2D and 3D viability is plotted (Fig 3B) and used to plot representative 2D viability assay data converted to 3D viability data (Fig 3C). There is a disparity between the conventional 2D viability assay and the 3D volume viability assay; the actual 3D viability is higher than that evaluated by the 2D area analysis. Using this conversion method, all 2D viability assay data were converted to the 3D islet viability data. A total 1278 human islets from four different donors were analyzed and plotted (Fig 3D). The oxygen levels of 100, 160, 270, 350 mmHg contained 355, 359, 328, and 236 islets data, respectively. Using this data, a second order curve of best fit was calculated for islet viability as a function of diameter (*d*) and media oxygen (*P*_*0*_). Coefficients with 95% confidence bounds are summarized in Table 2.

$$Viablity\left(d,P\right)=c_{00}+c_{10}\cdot P_{0}+c_{01}\cdot d+c_{20}\cdot P_{0}{}^{2}+c_{11}\cdot d\cdot P_{0}+c_{02}\cdot d^{2}$$ (7)

**Table 2:** Regression parameters for second order fit of viability data.

*Coefficient*	*Value*	*95% Confidence Bound*
c00	100	(98, 110)
c10	-0.015	(-0.049, 0.018)
c01	-0.018	(-0.034, -0.0023)
c20	-0.000017	(-0.000093, 0.000058)
c11	0.00021	(0.00017, 0.00026)
c02	-0.00013	(-0.00016, -0.00011)

This regression supports the idea that large islet diameter and low oxygen tension in the culture media synergistically induce low viability.

### Logistic regression model predicting the probability of islet survival

As a practical consideration, an estimation of oxygen tension required for islets survival based on isolated islet sizes is important for maintaining viable islets during culture. To perform this analysis, we defined an alive islet as one that has more than 95% 3D-viability, which corresponds to 86.4% 2D-viability (Fig 3C). A predicted survival probability of the isolated islets cultured in different oxygen tension level (Fig 3E) indicates that the survival rate declines in larger islets, whereas the survival improves by oxygenation, especially in the mid-sized (200–400 μm in diameter) islets. For example, islets 200 μm in diameter cultured in 50 mmHg show a 60.6% survival rate, whereas the viability of the same size islets improves to 95.3% when cultured in 350 mmHg oxygen.

## Discussion

Islet transplantation is an effective treatment for T1D. Islets are isolated from the pancreas of organ donors and transplanted into the liver by injection into the portal vein. However, there is still plenty of room to improve transplantation efficiency. One of the issues is the significant decrease of viable islet volume or islet number in the culture prior to transplantation [16, 34]. In addition, the hostile environment in the liver after transplantation may also reduce islet viability [10, 12, 35–37]. Another issue is that once islets are isolated from donor pancreata, their microenvironment drastically changes. The donor immune system no longer protects islets, and islets lose cell-cell communication, such as neuronal connection, to the native pancreata. Furthermore, they lose vascularization that is critical to deliver oxygen and nutrients. It is postulated that hypoxia plays an important role in islet loss in pre- and post-transplantation; however, information and knowledge are limited on islet physiology, especially in regards to oxygen requirements as well as toxicity. In the pancreas, the oxygen tension surrounding islets is reported as 30–40 mmHg [13, 38], and this level can increase close to the oxygen tension of the arterial blood (80–100 mmHg) because islets in the native pancreas contain a dense capillary network that provides abundant oxygen through blood circulation to all islet cells [39].

After the isolation process, isolated human islets are usually cultured under normoxic conditions that contain 21% oxygen, plus 5% CO_2_; this is the same culture condition used for most cells. Theoretically, the oxygen tension in culture medium under 21% oxygen at 1 atm is approximately 160 mmHg (760 mmHg × 21/100 = 160), which is far higher than that of the arterial blood. Cell death at the center of islets (central necrosis) occurs in many isolated islets under this oxygen setting. We previously reported that islet viability is well-maintained in culture under hyperoxic conditions, indicating that hyperoxic culture can alleviate central necrosis [16]. The central necrosis of isolated islets may be similar to that shown *in vivo* in tumors; tumors often show central necrosis caused by the imbalance between oxygen consumption by tumor cells and low oxygen supply [40]. This appears to be a distinctive feature of 3D structures consisting of many cells where an oxygen gradient exists [27]. Unlike large tumors, isolated islets are small; however, a 150 μm diameter islet contains an average of more than 2000 cells, with no systemic vascularity in the structure. Therefore, we hypothesized that the necrotic core in the isolated islet is caused by lack of oxygen. Oxygen simulations related to the islet were previously attempted based on the finite element method [22, 25, 26]; however, these studies mainly focused on the simulation itself. In the present study, we tested the hypothesis that lack of oxygen is one of the primary causes of central necrosis by determining the correlation between the hypoxic core revealed by the computational simulation and human islet viability assessed *in vitro*. These data provide a scientific basis to develop preventive measures to maintain viable isolated islets *in vitro* as well as *in vivo*.

As expected, the simulation data clearly demonstrate the oxygen gradient in average sized islets (150 μm in diameter) when cultured in 21% oxygen; only the first 30 μm shell is maintained in an oxygenated environment above 80 mmHg, which corresponds to the oxygen tension in the arterial blood. For this simulation analysis, several factors were needed including a single cell volume and OCR values (Table 1). We calculated a single cell volume using the islet sphere model (Fig 1A), assuming that the islet shape is a sphere and cells are uniformly distributed. Interestingly, the estimated single cell volume calculated using our method was similar to that calculated by another method previously published [15]. We did not calculate the volume differences among different cell types in the islet because the beta and alpha cells are the dominant cell types in the islet [29, 30].

We performed single cell OCR measurement using beta and alpha cell lines (Fig 1C) because preparation of single islet cells requires enzymatic digestion that damages the cells. There may be differences in OCR values between cell lines derived from tumor cells and native human islet cells. Because of the higher metabolism activity in tumor cells compared to primary cells, our simulation may overestimate the hypoxic condition in the islet core. However, the oxygen gradient and hypoxic core surely exist in the islet structure in any cases.

Another factor not included in this simulation is how the OCR change may be influenced by islet cell activity. It is well known that beta cells respond to high glucose to secrete insulin, which increases OCR [41, 42]. This microenvironmental change increases oxygen consumption and worsens the hypoxic condition inside the islet. Because microenvironmental changes occur when islets are transplanted into diabetic patients, the increase in the hypoxic environment should be considered after transplantation. In addition oxygen supply and consumption may be substantially changed by the establishment of angiogenesis in the *in vivo* islet transplantation model. These *in vivo*-specific microenvironmental changes are not considered in this *in silico* and *in vitro* study, which is a possible limitation for direct application of this study to simulations of *in vivo* oxygen in transplanted islets.

Because we demonstrated that hypoxia inside the islet structure caused islet cell death, hyperoxic culture should be a promising solution for providing sufficient oxygen deep into the islet core. During culture, cells in the islet core are exposed to the lowest oxygen tension. On the other hand, cells on the islet surface are exposed to the highest oxygen tension, identical to the surrounding environment. Once the oxygen level exceeds the non-toxic range, a large amount of cell volume on the surface should be damaged. For example, 95% oxygen increases the oxygen level inside large islets 300 μm in diameter, which increases the viability in the islet core. However, cells on the islet surface that are directly exposed to 95% oxygen are destroyed within 24 hours (Fig 2A). Therefore, islets should be cultured within the non-toxic range. The sensitivity to hyperoxia is known to be cell type-specific [32, 33, 43]; hence, we tested the non-toxic oxygen tension range applicable to islet culture. Small islets approximately 50 μm in diameter were used to test oxygen toxicity because oxygen tension is uniformly distributed in these islets and these cells do not undergo central necrosis in simulation (Fig 2A, right). As expected, dead cells in small islets distributed on surface, not in the core (Fig 2A, left). Higher than 75% oxygen is harmful to islet cells, causing significantly decreased islet viability (Fig 2B). In addition, FDA intensity (green fluorescence assay which measures intracellular esterase activity [28]) in 21% and 50% oxygen cultured islets was brighter than those cultured in higher oxygen tension, suggesting that cells cultured under these oxygen settings are metabolically active. Based on these results, 10% to 50% oxygen was used in the subsequent experiments to further simulate oxygen tension inside the islet structure under different oxygen tension in culture media (Fig 2C and 2D).

For *in silico* simulation under different oxygen environments, the absolute oxygen tension (mmHg) inside the well containing media and cultured islets was used for the simulation (Fig 2C and 2D). Media oxygen tension is not the same between the surface and bottom of the well [16], and this approach enabled us to precisely simulate the oxygen tension inside the islets. The dead cell volume in 300 μm diameter islets markedly decreased when cultured in a higher oxygen environment, whereas lower oxygen culture induced a larger volume of cell death in the islet core (Fig 2D). The simulated hypoxic core closely mimicked the experimental results measured *in vitro* (Fig 2C), and this agreement supports the hypothesis that hypoxia contributes to the development of central necrosis. In addition, viability was expressed as a function of both islet diameter and media oxygen to quantifiably describe the agreement. Coefficients of islet diameter (c01 and c02 in Table 2) are both negative, indicating that as islet diameter increases, viability decreases. Coefficients of media oxygen (c10 and c20 in Table 2) are both negative; however, it did not show the statistical significance because those confidence intervals include 0.

The logistic regression analyses for islet survival prediction clarified the oxygenated culture effect (Fig 3E). Isolated human islets are not uniform in size, generally ranging 50–500 μm in diameter. The volume of a 500μm diameter islet is 1000 times greater than that of a 50 μm diameter islet; therefore, cell death in large islets has great impact upon overall islet viability or viable islet cell volume [22]. This analysis clearly shows that the 21% oxygen culture commonly used for islets is not sufficient to alleviate cell death in large islets. The survival rate of 400μm diameter islets is 32.7% under 150mmHg culture compared to 73.1% under 350 mmHg culture. Those culture environments correspond to 21% and 50% oxygen culture of islets, both of which are proven to be safe for islet culture in terms of oxygen toxicity (Fig 2A and 2B).

Despite the improved viability and survival rates in oxygenated culture (Fig 3D and 3E), our analysis reveals that islet size is another limiting factor impacting how hypoxic core conditions affect the viability of islets. Our results are consistent with several studies that demonstrate the advantage of small islets on survival in experimental models as well as clinical islet transplantation studies [44–46]. In addition, small islets have improved insulin secretion function compared to large islets *in vitro* [47], which might be a result of higher oxygenation [16]. Because the size distribution of isolated islets is determined by the original islet size in the donor pancreas, selection of smaller islets prior to the transplantation may help eliminate the unfavorable effect of central necrosis in larger islets. Damaged cells in large islets are not only nonfunctioning but also harm other islets by releasing inflammatory cytokines. However, eliminating large islets also reduces the volume of beta cells available for transplantation.

Results shown in our *in vitro* study also implicate the reason for islet cell death after transplantation. Oxygen tension in the liver, where the islets engraft, is reported as 45–50 mmHg [48], and the implanted islets have to survive initially using diffused oxygen from surrounding blood and tissue until blood vessel formation established. Under the liver oxygen tension (50 mmHg), the survival rate of 100 μm islets is 80.8% and that of 400 μm islet is only 17% based on the survival regression analysis (Fig 3E). Because the survival rate of 400 μm islet nearly doubles in 150 mmHg compared to in 50 mmHg (32.7% vs. 17%), post-transplant oxygenation is a reasonable treatment to prevent such extensive islet death [48]. However, the oxygenation effect is limited for huge islets (>400 μm in diameter) (Fig 3E). Although the population of huge islets is low among isolated islets, the result indicates that oxygenation among the range tested cannot alleviate hypoxia-induced damage in those islets.

Lastly, the variability of human islets should also be taken into consideration. Unlike well-established animal models, the characteristics of human isolated islets are diverse, reflecting differences in donor backgrounds including age, sex, and race (Donors A-D in S1 Table). We calculated estimated survival rates for each donor islet in different oxygen environments (50, 150, 250, and 350 mmHg) (S1 Fig) and found that oxygen tolerance varied widely among islet donors. However, the islet survival rate was generally higher in high oxygen environments compared to those in low oxygen environments.

In summary, by comparing results from islet oxygen simulation and actual *in vitro* viability data, we demonstrated that oxygenation and islet size modulate the extent of central necrosis of isolated human islets. The effect of oxygenation on islet survival was significant. Therefore, we suggest that providing a hyperoxic environment is a promising approach to improving the outcome of pancreatic islet transplantation.

## Supporting information

S1 Table Donor information of the human islets in this analysis. (DOCX)

S2 Table Underlying data for figures. (XLSX)

S1 Fig Estimated survival rates of each donor islet in different oxygen environments. (PDF)
