# Supplementary material for: Oxygen environment and islet size are the primary limiting factors of isolated pancreatic islet survival
Source: PLoS One. 2017 Aug 23;12(8):e0183780. doi: 10.1371/journal.pone.0183780 (PMC5568442; doi:10.1371/journal.pone.0183780)
Supplement: S1 Table — (DOCX) [file pone.0183780.s001.docx]

**S1 Table. Donor information of the human islets in this analysis**

| Donor IDs | Sex | Race | Age | Weight (kg) | Height (m) | BMI | HbA1c% |
| --- | --- | --- | --- | --- | --- | --- | --- |
| A | M | White | 24 | 87.1 | 1.74 | 25 | 5.0 |
| B | M | White | 38 | 86.6 | 1.40 | 31 | 5.5 |
| C | M | Hispanic | 31 | 113.4 | 1.45 | 39 | 5.3 |
| D | M | White | 35 | 97.1 | 1.80 | 27 | 5.4 |
