# Supplementary figures and images for: Oxygen environment and islet size are the primary limiting factors of isolated pancreatic islet survival
Source: PLoS One. 2017 Aug 23;12(8):e0183780. doi: 10.1371/journal.pone.0183780 (PMC5568442; doi:10.1371/journal.pone.0183780)

pO<sub>2</sub> = 50 mmHg

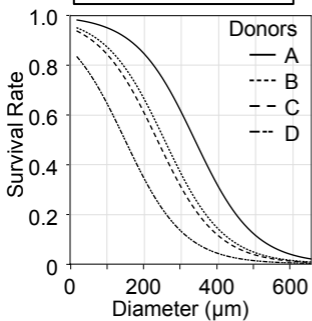

pO<sub>2</sub> = 150 mmHg

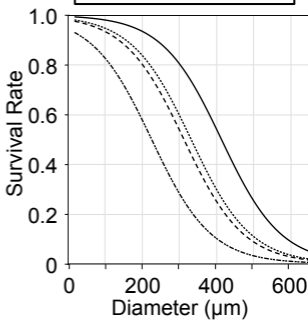

pO<sub>2</sub> = 250 mmHg

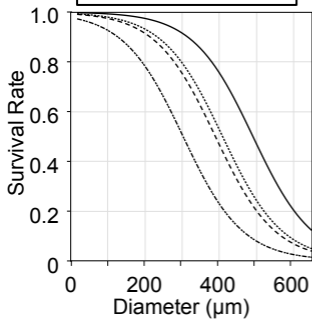

pO<sub>2</sub> = 350 mmHg

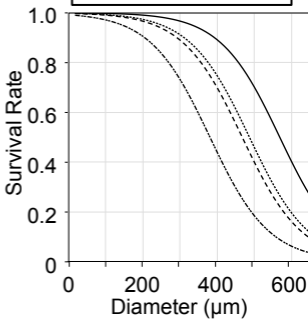

Supplement: S1 Fig — (PDF) [file pone.0183780.s003.pdf]
